# Partial Alignment of Electrocardiographic Subspaces Associated With Obesity and Heart Failure in the Third National Health and Nutrition Examination Survey

**DOI:** 10.7759/cureus.115932

**Published:** 2026-09-08

**Authors:** Patrick Dumitrescu, Haider Malik, Johnny Cobas Hernandez, Renato Gustavo Luque, Mariam Tkeshelashvili

**Affiliations:** 1 Internal Medicine Residency Program at Lee Health, Florida State University College of Medicine, Cape Coral, USA

**Keywords:** electrocardiography, heart failure, obesity, population health, principal angles, repolarization, subspace analysis

## Abstract

Background

Obesity and heart failure are each associated with electrocardiographic remodeling, but it is unknown whether their multidimensional electrical phenotypes share common directions within a feature space.

Methods

We conducted a cross-sectional analysis of historical Third National Health and Nutrition Examination Survey data (1988-1994) from 8,324 adults aged 40 years or older with complete data on obesity, self-reported physician-diagnosed heart failure, survey design variables, and resting 12-lead electrocardiography. Forty-seven features were adjusted for age, sex, and race/ethnicity, standardized, and transformed using covariance whitening relative to nonobese participants without heart failure. Across 30 repeated analyses that preserved the survey design and used one half of the sample, we estimated two-dimensional subspaces based on excess second moments. The obesity subspace was derived from obese participants without heart failure, and the heart failure subspace was derived from nonobese participants with heart failure. The primary statistic was the mean squared canonical cosine. Inference used a permutation test that repeated the full analytical pipeline in 5,000 iterations stratified by survey design.

Results

The cohort included 5,691 reference participants, 2,216 obese participants without heart failure, 287 nonobese participants with heart failure, and 130 participants with both conditions. The mean squared canonical alignment across the survey design splits was 0.205, compared with a permutation null mean of 0.088 and a 99th percentile of 0.120 (empirical p = 0.0002). Alignment was 0.216 after clinical adjustment, 0.219 when restricted to QRS durations below 120 ms, and 0.132 after excluding prior myocardial infarction and major conduction abnormalities. Full sample descriptive principal angles were 32.0 and 81.8 degrees. The leading shared direction was dominated by T-wave amplitudes and frontal T-axis features.

Conclusions

The electrocardiographic subspaces associated with obesity and self-reported physician-diagnosed heart failure demonstrated statistically nonrandom but incomplete alignment. One shared direction was dominated by repolarization features, while substantial structure remained unique to each survey-defined phenotype. These subspaces represent surface electrophysiological signatures of survey phenotypes, and the calculated principal angles are mathematical representations of historical data (1988-1994), rather than physiologically confirmed disease entities. They should not be interpreted as evidence of true physiological or subclinical heart failure mechanisms. These findings identify a target for longitudinal validation.

## Introduction

Obesity is a major risk factor for heart failure and is associated with altered loading conditions, ventricular remodeling, autonomic and metabolic perturbations, epicardial adiposity, and myocardial fibrosis [1,2]. These processes can influence cardiac activation and recovery before or alongside overt structural disease. Conventional studies have linked adiposity to changes in electrocardiographic voltages, intervals, axes, and repolarization measures [3-5], while artificial-intelligence models can infer body mass index and cardiometabolic risk directly from the 12-lead electrocardiogram [6].

Heart failure also has a multidimensional electrical phenotype. Axes, QRS-T relationships, conduction measures, and repolarization abnormalities have been associated with prevalent and incident heart failure [7-9]. Standard regressions generally test one marker at a time, however, and black-box prediction models do not quantify whether remodeling related to obesity and heart failure occupies shared directions in an interpretable feature space.

We therefore represented obesity and heart failure as low-dimensional geometric phenotypes. For each condition, we estimated directions of excess electrocardiographic variation relative to a common reference group without obesity or heart failure and used principal angles to quantify subspace alignment [10,11]. We hypothesized that the two subspaces would demonstrate partial, statistically nonrandom alignment, consistent with one shared electrical component and additional structure unique to each condition.

## Materials and methods

Study design and population

We conducted a cross-sectional secondary analysis of the Third National Health and Nutrition Examination Survey (NHANES III, 1988-1994), a nationally representative survey of the noninstitutionalized United States population [12]. Standard resting 12-lead electrocardiograms were obtained in mobile examination centers from sampled adults aged 40 years or older [13,14]. Public-use questionnaire, examination, survey design, and electrocardiography files were merged by participant sequence number. The overall NHANES III examination response rate was 78%. Among examined adults, electrocardiography completion rates were 96% at ages 40-59 years, 95% at ages 60-74 years, and 91% at ages 75 years or older [12-14].

Among 8,561 electrocardiographic records, 17 participants were excluded because of unknown self-reported heart failure status, 14 because of unknown measured obesity status, and 206 because of incomplete required core ventricular electrocardiographic measurements. No additional participants were excluded for missing survey-design or adjustment variables or for values outside the prespecified physiologic limits of a heart rate of 30-220 beats/min, QRS duration of 50-220 ms, and Fridericia-corrected QT interval of 250-650 ms. The analytic sample contained 8,324 participants, representing 97.2% of the available electrocardiographic records. Although examination weights incorporated NHANES III nonresponse adjustments, residual selection bias remains possible if participation or electrocardiography availability was associated with characteristics not fully represented by those weights.

Phenotypes and electrocardiographic feature space

Obesity was defined as a measured body mass index of at least 30 kg/m². Heart failure was defined solely by an affirmative response to the survey question asking whether a physician had ever diagnosed congestive heart failure. No medical-record adjudication, cardiac imaging, or biomarker confirmation was available. “Heart failure” therefore denotes a self-reported survey phenotype rather than a physiologically confirmed disease entity. A previous validation study found high specificity but limited sensitivity for self-reported heart failure compared with physician-diagnosed heart failure [15]. Four mutually exclusive groups were formed: nonobese participants without heart failure (reference), obese participants without heart failure, nonobese participants with heart failure, and obese participants with heart failure. The obesity subspace was estimated only from obese participants without heart failure, and the heart failure subspace was estimated only from nonobese participants with heart failure. This separation reduced automatic overlap caused by obesity within the heart failure group.

The 47-feature space included heart rate, QRS duration, Fridericia-corrected QT interval, sine and cosine encodings of the frontal QRS and T axes, and lead-level R-wave, S-wave, J-point, negative T-wave, and positive T-wave amplitudes from leads I, II, and V1-V6. Circular encoding made axis values near −180 and +180 degrees adjacent. Quality rules were defined without reference to condition labels; the prespecified retention threshold was no more than 10% missingness and at least six distinct observed values. Among the 47 retained electrocardiographic features, feature-level missingness ranged from 0% to 0.565%. Overall, 8,255 participants (99.17%) had complete data for all 47 features, while 69 (0.83%) had at least one missing feature. The most frequent patterns involved jointly missing V2/V3 measurements (17 participants) or V1/V3 measurements (16 participants). Missingness for clinical sensitivity variables did not exceed 1.17%.

Survey design preprocessing and subspace estimation

Analyses incorporated the NHANES III mobile examination center examination weights and the public pseudo-strata and pseudo-primary sampling units (PSUs). For each of 30 prespecified random seeds, the 98 PSUs (2 in each of 49 pseudo-strata) were partitioned into complementary half-samples by randomly selecting one entire PSU per stratum for the estimation half and assigning the other PSU to the complementary half; participants within a PSU were never divided. The original examination weights were carried with participants without rescaling. All weighted medians, regressions, covariances, and second moments were ratio estimators normalized by the sum of weights in the selected half, so multiplying all half-sample weights by the conventional factor of two would not change the preprocessing parameters or subspace bases. Each estimation half therefore retained the original relative unequal selection and nonresponse weighting, included one PSU from every pseudo-stratum, and respected PSU clustering. This balanced repeated half-sample strategy was selected because the estimands depend on weighted moments and covariances rather than individual-level prediction loss; it permits estimation across the complete set of strata while assessing sensitivity to which PSU contributes to estimation. The repeated split range was used only as a stability summary and not as a design-based standard error or confidence interval.

Within reference-group participants in each estimation half, missing values were replaced by weighted medians; weighted median imputation was selected because feature missingness was sparse and to avoid further reducing the smaller heart failure groups. The missingness mechanism could not be empirically classified as missing completely at random, missing at random, or missing not at random. Median imputation may attenuate feature variability, and bias remains possible if missingness depended on unobserved values. Continuous features were winsorized at the weighted 0.5th and 99.5th percentiles; weighted models removed expected effects of age, age squared, sex, and race/ethnicity; and residuals were standardized to the variation in the reference group. The standardized residual matrix was transformed using covariance whitening based on the reference covariance matrix, so Euclidean geometry in the transformed space approximated Mahalanobis geometry in the residual feature space.

Within each estimation half, weighted second-moment matrices were calculated for the reference group, obese participants without heart failure, and nonobese participants with heart failure. Subtracting the reference second moment yielded a contrast matrix for each condition. The leading two positive eigenvectors of each contrast matrix defined two-dimensional obesity and heart failure subspaces, conceptually related to contrastive principal-component analysis [10]. Two dimensions were fixed for the final inferential pipeline.

For orthonormal two-dimensional bases VO and VH, singular value decomposition of VOᵀVH yields canonical cosines cos(θ1) and cos(θ2), where θ1 and θ2 are the principal angles [11]. The primary statistic was the average of cos²(θ1) and cos²(θ2). Values near zero indicate orthogonal subspaces, and values near one indicate coincident subspaces. This index is not a causal proportion or a variance-explained estimate. We report its mean over 30 splits and the empirical 2.5th-97.5th percentile split range as a stability summary, not a confidence interval.

Permutation inference, sensitivity analyses, and interpretation

Primary inference used 5,000 permutations conditional on the observed survey design and the 30 fixed PSU half-sample assignments. Within each estimation half, heart failure labels were shuffled separately within cells defined by obesity status and pseudo-stratum; because each half contained one intact PSU per stratum, permutations occurred only among participants in that selected PSU and preserved the observed heart failure count in every obesity-by-stratum cell. Examination weights, stratum and PSU identifiers, and split membership were held fixed. For every permutation, the complete analytical pipeline was repeated, including definition of the permuted-label reference group, imputation, winsorization, weighted residualization and standardization, whitening, and estimation of both subspaces. The null statistic was the mean alignment across the same 30 splits. This conditional strategy preserves the weighted sample composition, cluster membership, and stratum restrictions relevant to the estimator while testing label exchangeability within the specified design and obesity cells; re-estimation of the entire pipeline also propagates label-induced variation in preprocessing and both subspaces. The empirical upper-tail p value was calculated by adding one to both the number of permuted statistics at least as large as the observed statistic and the total number of permutations. It is a design-respecting conditional permutation p value, not a Taylor-linearized or replicate-weight design-based p value.

Sensitivity specifications added smoking, hypertension, diabetes, and coronary disease to normative adjustment; restricted QRS duration to below 120 ms; excluded prior self-reported or electrocardiographically probable myocardial infarction and major ventricular conduction abnormalities; and combined these exclusions with clinical adjustment. These specifications did not receive separate permutation tests. For descriptive interpretation only, subspaces were re-estimated in the full sample. Rotation-invariant feature participation was the sum of squared basis coefficients across the two dimensions. Shared canonical coefficients were projected back through the whitening transformation to the residual feature scale. Because canonical signs are arbitrary, absolute products were emphasized.

Ethics, reporting, software, and artificial-intelligence assistance

The source survey protocols received institutional review approval from the National Center for Health Statistics, and participants provided documented informed consent [12]. The present analysis used publicly available, deidentified data without participant contact. The Lee Health Institutional Review Board determined that this secondary analysis qualified for exemption under Category 4, 45 CFR 46.104(d)(4). Reporting was guided by the Strengthening the Reporting of Observational Studies in Epidemiology statement [16].

The analyses were performed in Google Colaboratory using Python version 3.12.13 (Python Software Foundation, Wilmington, DE, USA). Figures were generated with Matplotlib version 3.10.0 (Matplotlib Development Team; https://matplotlib.org). OpenAI ChatGPT was used during manuscript preparation to assist with language organization, code review, numerical-consistency checks, and drafting Python code for the numerical graphs. The figures are deterministic plots generated from numerical results; no generative-image model was used. The authors reviewed all outputs and remain responsible for the code, numerical results, references, interpretation, and submitted text.

## Results

Participant characteristics

The analytic sample included 5,691 nonobese participants without heart failure, 2,216 obese participants without heart failure, 287 nonobese participants with heart failure, and 130 participants with both conditions (Table 1). Participants with heart failure were older and had higher prevalences of hypertension, diabetes, prior myocardial infarction, and major ventricular conduction abnormalities than the reference group.

**Table 1:** Survey-weighted characteristics of the analytic population Values are presented as unweighted n or survey-weighted estimates with 95% confidence intervals. BMI, body mass index; HF, heart failure; MI, myocardial infarction; QTcF, Fridericia-corrected QT interval

Characteristic	Reference BMI <30, no HF	Obesity without HF BMI ≥30, no HF	HF without obesity BMI <30, HF	Obesity and HF BMI ≥30, HF
Unweighted n	5691	2216	287	130
Age, years	56.7 (55.8–57.5)	55.8 (55.0–56.7)	69.4 (67.3–71.6)	61.1 (58.8–63.5)
Female	52.3% (50.8–53.8)	57.3% (54.8–59.9)	46.8% (37.0–56.6)	51.9% (37.9–65.9)
Body mass index, kg/m²	24.7 (24.6–24.9)	34.4 (34.1–34.7)	25.4 (24.8–26.0)	34.7 (33.4–35.9)
Waist circumference, cm	90.2 (89.8–90.7)	111.1 (110.3–111.8)	93.4 (91.3–95.5)	113.1 (110.0–116.3)
QRS duration, ms	97.8 (96.7–98.9)	98.8 (97.7–99.9)	101.2 (98.1–104.4)	104.0 (100.4–107.5)
QTcF, ms	421.0 (419.9–422.2)	424.2 (423.1–425.4)	426.7 (422.6–430.9)	428.5 (421.3–435.6)
Physician-reported hypertension	25.8% (24.3–27.3)	49.9% (46.3–53.5)	52.9% (44.0–61.7)	80.5% (68.4–92.6)
Physician-reported diabetes	5.6% (4.9–6.3)	13.1% (11.0–15.2)	18.1% (11.9–24.3)	30.7% (20.4–41.0)
Current cigarette smoking	25.0% (22.9–27.0)	18.3% (16.1–20.6)	23.0% (11.5–34.5)	25.0% (11.3–38.6)
Self-reported MI or ECG probable MI	3.8% (3.0–4.6)	4.4% (3.2–5.6)	58.9% (50.7–67.2)	60.5% (47.6–73.4)
Major ventricular conduction abnormality	4.9% (3.7–6.0)	6.1% (4.4–7.9)	12.4% (6.1–18.8)	12.5% (5.1–20.0)

Primary subspace alignment

The primary mean squared canonical alignment across the survey design splits was 0.20485. The permutation null mean was 0.08756, with 95th and 99th percentiles of 0.11052 and 0.12036. None of the 5,000 permuted statistics equaled or exceeded the observed statistic, yielding p = 0.0002 (Figure 1). Across the 30 splits, the alignment stability range was 0.031-0.380. The mean minimum principal angle was 53.0 degrees (split range, 34.3-75.8), and the mean of the two angles was 67.5 degrees (56.3-82.3).

**Figure 1:**
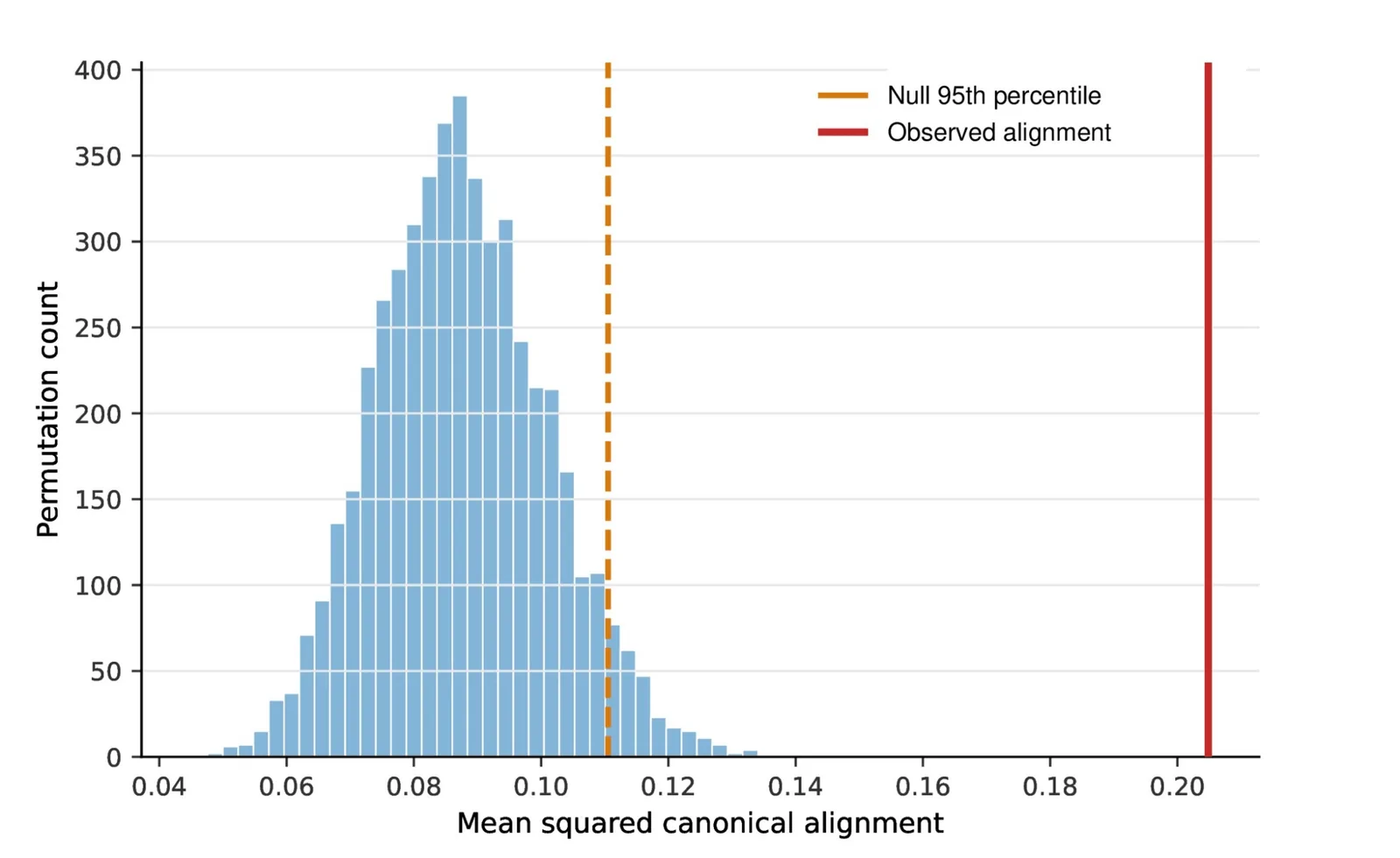
Full-pipeline permutation distribution for obesity–heart failure electrocardiographic subspace alignment The observed statistic was 0.205; the null mean was 0.088, and the null 95th percentile was 0.111. All preprocessing and both subspaces were re-estimated in every permutation. The empirical upper-tail p value was 0.0002.

Sensitivity analyses

Alignment was similar after clinical covariate adjustment (0.216) and restriction to QRS durations below 120 ms (0.219). It was smaller after excluding prior myocardial infarction and major ventricular conduction abnormalities (0.132) and in the strict-exclusion plus clinical-adjustment specification (0.149) (Table 2 and Figure 2). These sensitivity ranges are split stability summaries and do not provide independent hypothesis tests.

**Table 2:** Design-split alignment in primary and sensitivity analyses Values are means with empirical 2.5th–97.5th percentile ranges over 30 design-respecting half-sample analyses. Ranges are stability summaries, not confidence intervals. The primary permutation p value does not apply to sensitivity specifications. MI, myocardial infarction

Specification	Analytic n	Mean squared canonical alignment	Minimum principal angle, degrees	Mean principal angle, degrees	Permutation p value
Primary analysis	8324	0.205 (0.031–0.380)	53.0 (34.3–75.8)	67.5 (56.3–82.3)	0.0002
Additional clinical covariate adjustment	8324	0.216 (0.029–0.391)	52.6 (32.7–76.5)	66.5 (51.6–81.7)	Not tested
QRS duration <120 ms	7792	0.219 (0.069–0.369)	50.8 (31.8–68.8)	66.1 (56.7–76.7)	Not tested
Excluded prior MI and major ventricular conduction abnormality	7175	0.132 (0.027–0.227)	60.9 (48.7–77.4)	72.4 (65.0–81.5)	Not tested
Strict exclusions plus clinical adjustment	7167	0.149 (0.025–0.283)	59.4 (42.2–79.0)	71.0 (59.5–83.0)	Not tested

**Figure 2:**
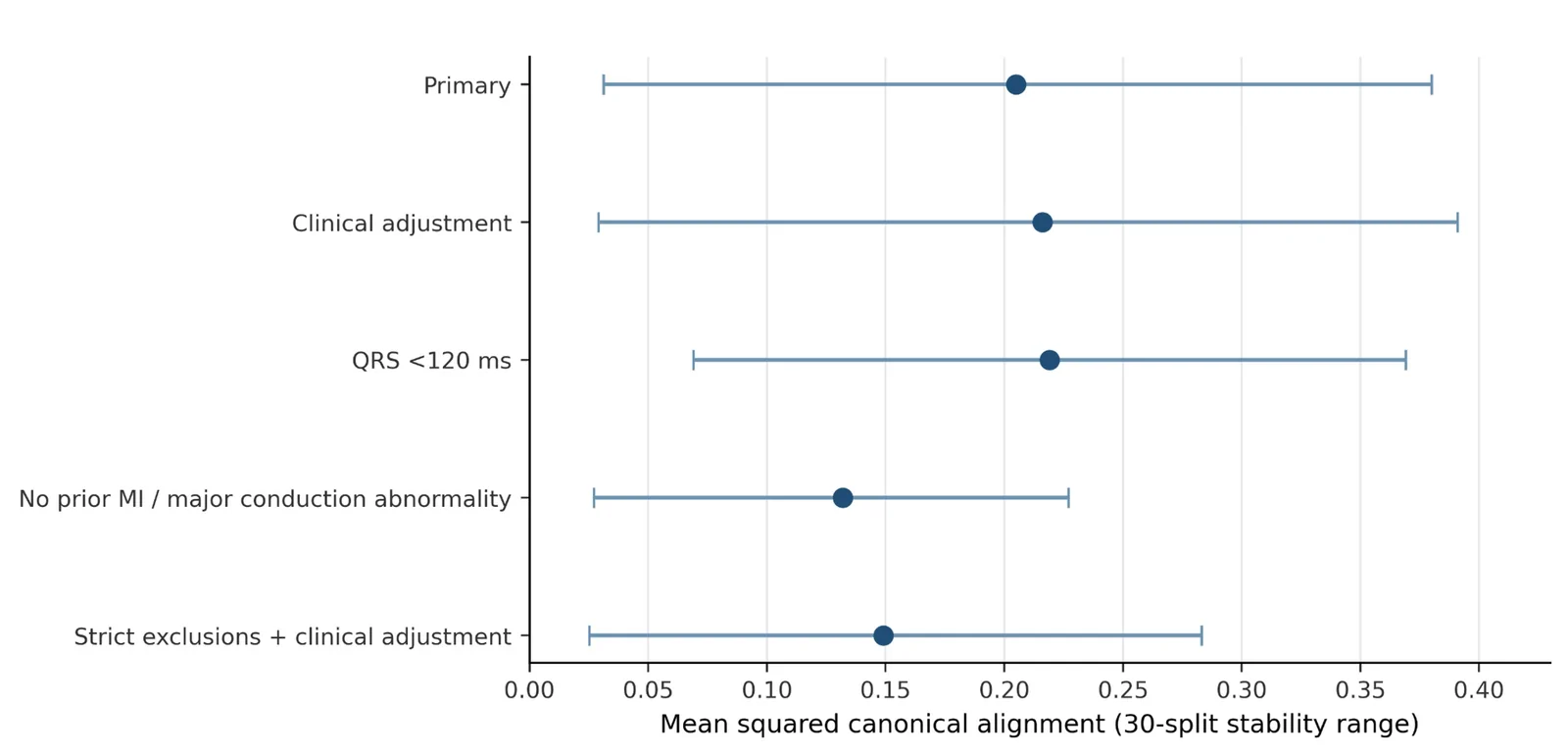
Primary and sensitivity estimates Points are means, and horizontal bars are empirical 2.5th–97.5th percentile ranges across 30 splits. Ranges are stability summaries, not confidence intervals. Only the primary specification received a permutation test. MI, myocardial infarction

Full sample descriptive geometry

In the full-sample descriptive analysis, the principal angles were 31.96 and 81.81 degrees, with canonical cosines of 0.848 and 0.142 and a mean squared alignment of 0.370. The leading shared direction was dominated by negative T-wave amplitude in lead V4, negative T-wave amplitude in leads I and II, positive T-wave amplitude in lead V5, and frontal T-axis cosine (Table 3 and Figure 3). The nearly orthogonal second direction indicated substantial structure unique to each condition.

**Table 3:** Leading features in the first full-sample shared canonical direction Participation and contributions are descriptive; canonical signs are arbitrary. HF, heart failure

Feature label	Obesity subspace participation	HF subspace participation	Shared canonical contribution
Negative T-wave amplitude, lead V4	0.0333	0.0682	0.1400
Negative T-wave amplitude, lead I	0.0299	0.0862	0.1148
Negative T-wave amplitude, lead II	0.0496	0.0381	0.1147
Positive T-wave amplitude, lead V5	0.0776	0.0655	0.0848
Frontal T-axis cosine	0.0758	0.0361	0.0808
Positive T-wave amplitude, lead I	0.0792	0.0075	0.0502
Negative T-wave amplitude, lead V6	0.0093	0.2343	0.0428
Frontal T-axis sine	0.0271	0.0324	0.0414
J-point amplitude, lead V4	0.0057	0.0231	0.0400
Positive T-wave amplitude, lead V4	0.0081	0.0332	0.0347
Positive T-wave amplitude, lead V6	0.0391	0.0237	0.0271
Negative T-wave amplitude, lead V3	0.0134	0.0076	0.0252
J-point amplitude, lead V2	0.0115	0.0086	0.0240
R-wave amplitude, lead V5	0.1326	0.0210	0.0235
J-point amplitude, lead V3	0.0153	0.0237	0.0204

**Figure 3:**
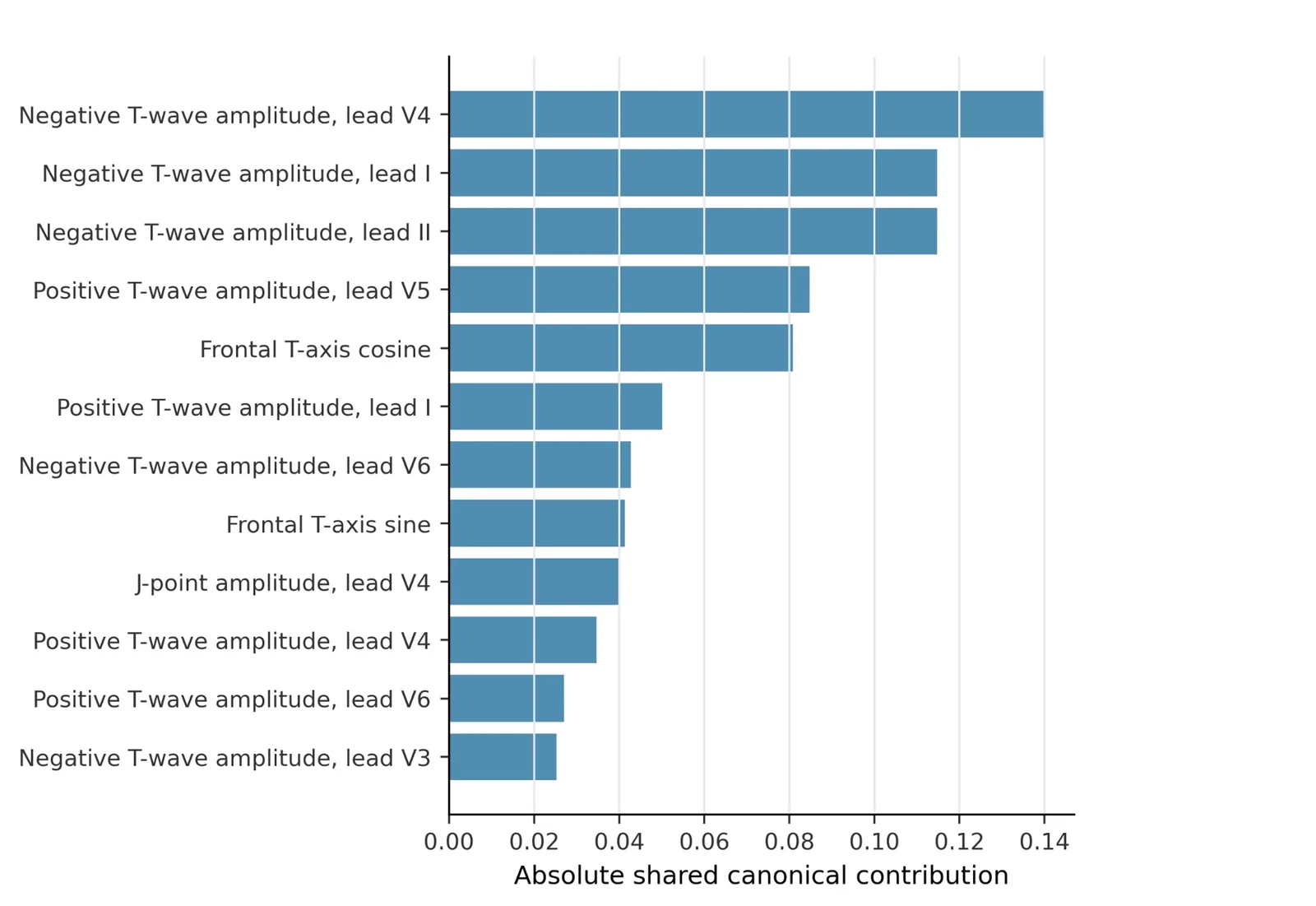
Absolute products of obesity and heart-failure shared canonical coefficients for the 12 leading features Interpretation is descriptive and based on the full analytic sample.

## Discussion

In this nationally representative cross-sectional sample, the two-dimensional electrocardiographic subspaces associated with obesity and self-reported physician-diagnosed heart failure demonstrated statistically nonrandom but incomplete alignment. The observed statistic across the survey design splits was more than twice the permutation null mean, but its absolute value was 0.205, and one canonical pair in the full sample was nearly orthogonal. These findings describe mathematical alignment between surface electrocardiographic signatures of survey-defined phenotypes and are inconsistent with a single continuum in which obesity reproduces the complete electrocardiographic signature associated with the self-reported heart failure label.

The leading shared direction was dominated by repolarization features. Prior studies have associated obesity with QT, T-wave, voltage, and axis changes [3-6], while QRS-T and T-axis abnormalities have been linked to the risk of heart failure [7-9]. This analysis extends those observations from individual markers to joint geometry. Principal angles mathematically distinguish a shared direction from directions unique to each survey-defined phenotype without asserting prediction, mediation, causality, or a physiological heart failure mechanism.

The smaller estimate after excluding prior myocardial infarction and major conduction abnormalities suggests that established ischemic or conduction pathology contributes to the observed overlap. In contrast, restriction to QRS durations below 120 ms produced a result similar to the primary analysis. Body habitus and cardiac orientation can alter surface amplitudes and axes [17], so the shared subspace should be interpreted as a surface electrocardiographic phenotype, not direct evidence of a particular myocardial mechanism. Linking this direction to imaging measures of obesity-related remodeling will be essential [18].

Contemporary electrophysiological and structural remodeling may also be influenced by dynamic exposures that were absent from NHANES III. In patients with pre-existing heart failure, post-acute COVID-19 has been associated with persistent myocardial inflammation, microvascular endothelial dysfunction, autonomic and neurohormonal dysregulation, and adverse heart failure trajectories [19]. These processes may contribute to ventricular depolarization-repolarization heterogeneity, including T-wave and axis changes relevant to the repolarization features summarized in Table 3. Because NHANES III was conducted in 1988-1994, this evidence provides contemporary pathophysiological context only and cannot establish that post-viral mechanisms produced the historical subspace observed in this study.

Clinical interpretation

The practical interpretation is conservative. Most electrical structures associated with heart failure did not align with the obesity subspace. Therefore, an abnormal electrocardiogram in a person with obesity should not be interpreted as evidence of subclinical heart failure based on this study. The shared direction instead supplies a focused target for future longitudinal work: investigators can test whether movement along this repolarization-dominated axis precedes imaging abnormalities or adjudicated heart failure.

Strengths and limitations

Strengths include standardized electrocardiography, a population-based sample, survey-weighted preprocessing, separation of the two derivation groups, covariance whitening, repeated estimation using survey design splits, and a permutation test that repeated the complete analytical pipeline 5,000 times. Repeating all preprocessing during permutation reduces the chance that statistical significance arises from fixed, label-informed transformations.

The analysis is cross-sectional and cannot establish temporal order or causality. Heart failure status was based on self-report of a prior physician diagnosis and was not independently adjudicated. Recall error, misunderstanding of the questionnaire, differences in diagnostic access, or confusion with other causes of dyspnea or edema could have produced false-positive and false-negative classification. Nondifferential misclassification would generally attenuate group contrasts, whereas differential misclassification related to obesity, health-care use, or comorbidity could shift the estimated heart failure subspace in an unpredictable direction. Ejection fraction, cardiac imaging, natriuretic peptide concentrations, and adjudicated clinical records or heart failure subtype information were unavailable; therefore, heart failure with preserved versus reduced ejection fraction and other etiologic subtypes could not be distinguished, and the heart failure group may represent a heterogeneous mixture of syndromes. The reported ECG subspace should accordingly be interpreted as the electrical pattern associated with self-reported physician-diagnosed heart failure in this survey, not as a validated heart failure signature, a mechanism-specific remodeling phenotype, or a subtype-specific marker.

The overall NHANES III examination response rate was 78% [12]. Although examination weights incorporated adjustments for survey nonresponse, residual selection bias remains possible if examined and nonexamined adults differed in ways not captured by the weighting variables. The missingness mechanism could not be definitively classified as missing completely at random, missing at random, or missing not at random. Although electrocardiographic-feature missingness was low, weighted median imputation does not account for imputation uncertainty and may attenuate feature variability; bias remains possible if missingness was informative.

The survey is historical (1988-1994) and predates contemporary therapies, including sodium-glucose cotransporter-2 inhibitors, angiotensin receptor-neprilysin inhibitors, and glucagon-like peptide-1 receptor agonists, which may alter structural, metabolic, and electrical cardiac remodeling. Accordingly, the calculated principal angles are analysis-dependent mathematical representations of self-reported survey labels rather than measurements of physiologically confirmed disease entities, and their applicability to contemporary clinical practice is uncertain. The heart failure group was also much smaller than the reference and obesity groups. Variability among the splits was substantial; the reported ranges are not design-based confidence intervals. Surface amplitudes are affected by body habitus and acquisition factors. No independent cohort was available. The full-sample feature coefficients are descriptive. Two dimensions were fixed for the final inferential pipeline, so uncertainty regarding the number of dimensions remains for future validation.

## Conclusions

Obesity and self-reported physician-diagnosed heart failure exhibited statistically nonrandom but incomplete alignment of their multidimensional surface electrocardiographic phenotype subspaces. One shared direction was dominated by ventricular repolarization features, whereas the second direction was largely distinct.

These findings provide an interpretable framework for separating shared surface electrocardiographic signatures from structure unique to each self-reported survey phenotype. The calculated principal angles are analysis-dependent mathematical representations derived from historical NHANES III data, rather than physiologically confirmed disease entities, and their applicability to contemporary clinical practice with modern therapies is uncertain. They do not establish true physiological or subclinical heart failure mechanisms or a longitudinal transition from obesity to heart failure and should not guide interpretation of an individual patient’s electrocardiogram. External validation using adjudicated heart failure, imaging, and longitudinal electrocardiography is required.
